# An Insight into Cancer Cells and Disease Progression Through the Lens of Mathematical Modeling

**DOI:** 10.3390/cimb47070477

**Published:** 2025-06-20

**Authors:** Polychronis Michalakis, Dimitra Vasilaki, Ali Jihad Abdallah, Charilaos Asikis, Athina Niakou, Athanasios Stratos, Alexandros Tsouknidas, Elaine Johnstone, Konstantinos Michalakis

**Affiliations:** 1 Faculty of Health Sciences, European University of Cyprus, Nicosia 2404, Cyprus; paulmichalakis@hotmail.com; 2 Center for Multiscale and Translational Mechanobiology, School of Dental Medicine, Boston University, Boston, MA 02118, USA; dvasilak@bu.edu (D.V.); aja92@bu.edu (A.J.A.); asikis@bu.edu (C.A.); atsouk@bu.edu (A.T.); 3 Faculty of Health Sciences, Aristotle University of Thessaloniki, 54124 Thessaloniki, Greece; aeniakou@dent.auth.gr (A.N.); astrato@dent.auth.gr (A.S.); 4 Nuffield Department, University of Oxford, Oxford OX3 7BN, UK

**Keywords:** cell biomechanics, cancer biomechanics, DNA mechanics, cell organelles mechanical properties, cancer initiation, cancer progression, mathematical modeling

## Abstract

During cancer initiation, normal cells acquire mutations disrupting standard cellular processes, activating oncogenes and inactivating tumor suppressor genes, acquiring the well-described hallmarks of cancer on the path to malignancy. This process is influenced by a combination of physiological and metabolic pathways, as well as environmental cues, and leads to abnormal cell cycle, increased cell motility, and invasive characteristics. Cancer cell organelles also present some distinct differences from those of normal cells. Cancer progression requires certain tumorigenic biochemical pathways to be activated. However, mechanical cues are also important, as they have an effect on cell differentiation and fate. A continuous biochemical–biomechanical interaction exists, which affects the mechanical properties of the cells, as well as their behavior. This review aims to focus on the mathematical relationships governing cancer mechanobiology and examine how the altered mechanical properties of a cancer cell may affect malignant progression.

## 1. Introduction

Cancer represents a disease with extreme heterogeneity. Malignant tumors in different tissues display strikingly dissimilar behaviors, response to therapeutic interventions, and prognoses. It is well-known that tumors arising in certain organs, e.g., the pancreas, are extremely aggressive and have a poor prognosis, while others, such as those found in the bladder (non-muscle invasive bladder cancer), have a 96% 5-year survival rate [1,2].

It is well recognized that cancer initiation and progression involves many tumor-promoting genes, many of which are also tissue-dependent [3]. The catalog of somatic mutations in cancer (COSMIC) pioneered a high-quality gene census resource which provides information on millions of mutations across thousands of cancer types [4]. This research has indicated that mutations in more than 1% of the human genes contribute to tumorigenesis. Until the beginning of the 21st century, only 291 cancer genes had been identified [5]. Genes that are commonly mutated are associated with cell proliferation, apoptosis, chromatin regulation, genome stability, immune evasion, RNA processing, and protein homeostasis [6].

Research in cancer specimens has also demonstrated thousands of upregulated or downregulated genes [7,8,9]. However, it is not known yet which are the specific transcriptionally deregulated genes that play an instrumental role in cancer initiation and progression, and which genes are the bystanders [3].

Although tumorigenic biochemical pathways are essential in cancer initiation and progression, studies have shown that mechanical cues are also important, as they have an effect on cell differentiation and fate [10,11]. It has been demonstrated in the past that there is a continuous biochemical–biomechanical interaction, which directly affects cell behavior [12,13,14,15]. Τhere are multiple stimuli that contribute to generating those changes that then result in the neoplastic cell. Among the initial stimuli implicated in neoplastic transformation, inflammation is considered fundamental, as it can induce biochemical alterations that trigger tumor-promoting processes. As mutations and cancer initiation deregulate the mechanobiochemical homeostatic equilibrium, the mechanical properties of the cells and the tissues involved change [16]. Moreover, biomechanical factors, such as stiffness, ECM composition, or mechanical stress, are involved in tumor progression, as cancer cells interact with neighboring cells and the extracellular matrix (ECM). There is scarce information in the literature on mathematical modeling and the biomechanical response of cancer cell organelles. The purpose of this review is to focus mainly on the mathematical modeling of cancer mechanobiology and investigate how the altered mechanical properties of a cancer cell may affect disease progression. However, it is important to acknowledge that research in cancer mechanobiological modeling is still novel, presenting numerous unresolved challenges and limitations, especially concerning the comparative analysis of diverse modeling approaches.

## 2. Genetic Alteration of Cell

**Figure 1:**
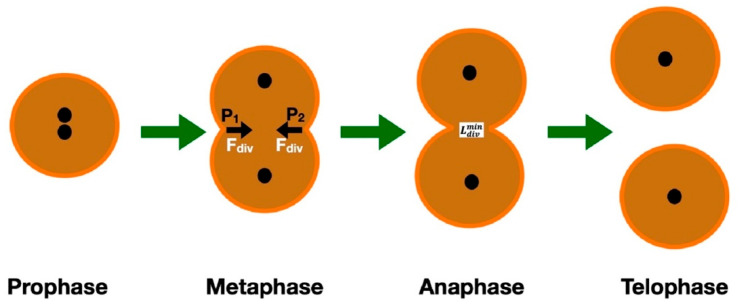
The 4 different phases of cancer cell mitosis. Note the contractile forces at points P_1_ and P_2_ in the metaphase, as well as the minimum distance required for splitting into two cells, which occurs in the anaphase.

It is generally accepted that a tumor arises from one genetically altered cell, which accumulates several mutations over a prolonged period [17,18]. This cell does not conform to the growth-controlling mechanisms that normal cells obey. It grows fast and changes its microenvironment by developing gradients of key metabolites such as glucose, oxygen, and growth factors. These gradients are a result of the tumor’s rapid growth and inefficient vasculature, leading to regions of hypoxia and nutrient deprivation. This metabolic reprogramming, including the preference for glycolysis even in the presence of oxygen (Warburg effect), supports cancer cell survival and proliferation in a challenging microenvironment [19,20]. These biochemical changes affect both the survival and the development of cancer cells [21]. Moreover, it has been shown that cancer cell growth is also influenced by differentiated signaling pathways, which have resulted from mutations and are activated during the process. In a hypoxic environment, common in rapidly growing tumors, cell metabolism changes and overridden apoptotic pathways also seem to be essential for both the survival and proliferation of cancer cells [22,23]. When a cancerous parent cell becomes mature and reaches a proliferative size, it may split into two cells through the process of mitosis. It is known that tumor progression is characterized by abnormal cell proliferation, due to the altered expression of proteins related to the cell cycle. A mathematical modeling of the contractile forces applied to the cancer cell during the metaphase, as well as the minimum length between the opposing walls of the plasma membrane for the division to occur, is given by the following [19] (Figure 1):

(1) $$F_{div}(l,\;t)=F_{div}\frac{\left\|P_{1}(t)-P_{2}(t)\right\|-L_{div}}{\left\|P_{1}(t)-P_{2}(t)\right\|}(P_{1}(t)-P_{2}(t))$$

and

(2) $$\left\|P_{2}(t)-P_{1}(t)\right\|\geq L_{div}^{min}$$

where $F_{div}$ are the contractile forces applied on diametrically opposed points P_1_ and P_2_, $F_{div}$ represents the constant spring stiffness, $L_{div}$ is the spring’s constant resting length, and $L_{div}^{min}$ represents the minimum length between the opposing walls of the plasma walls for cell division. When $L_{div}^{min}$ is reached, then the opposing contractile forces applied to points P_1_ and P_2_ cease, and the cancer cell divides into two daughter cells. The latter have certain features that warrant survival beyond a normal cell’s life cycle, as well as different morphological characteristics from those of normal cells. Following the mitosis, the two daughter cells start growing until they reach the proliferative size, and the whole process is usually replicated.

However, cancer progression is largely affected by biophysical processes and biochemical interactions, including the availability of nutrients. Τhis is fundamental, because it guarantees tumor growth and simultaneously impoverishes the organism, which gradually progresses from a state of malnutrition to sarcopenia and ultimately to cachexia [24,25]. An important finding is that cancer cells have a hypoxic phenotype, which makes them able to progress in low-oxygen conditions [26]. This progression takes place because cancer cells adjust to anaerobic metabolism. Moreover, other properties associated with hypoxia include resistance to apoptosis, epithelial–mesenchymal transition, anti-cancer treatment persistence, inflammation, genomic instability, and Vascular Endothelial Growth Factor (VEGF) release, which promotes tumor angiogenesis [22,26].

As a cancer cell divides and the process repeats itself, forces are generated. Mathematically, a single tumor cell can be thought to be a spheroid elastic body surrounded by an incompressible viscous fluid, which can be either a non-tumor tissue or the ECM [19,24,27,28]. In most of the current models, this fluid has a viscosity of μ = 100 g/(cm·s). The cytoplasm is also modeled as fluid. However, as the cytoskeleton and the extracellular fibers are usually omitted, a fluid of higher viscosity is modeled, for compensation reasons [27]. The motion of the fluid in this model is governed by the Navier-Stokes equations, describing the balance of momentum and that of mass in an incompressible viscous fluid, with distributed sources [29,30]:

(3) $$\rho \left(\frac{\partial u}{\partial t}+\left(u\cdot \nabla \right)u\right)=-\nabla p+\mu \Delta u+\frac{\mu }{3\rho }\nabla +f$$

(4) p∇·u=s

where ρ represents the constant fluid density, u is the fluid velocity, t is the time, p is the fluid pressure, μ is the constant fluid viscosity, f is the external force density, ∇·u represents the local rate of fluid expansion, and s is the fluid source distribution. In this model, the fluid is generally considered to be incompressible everywhere, with the exception of some discrete points, at which the cell growth occurs. Rejniak has postulated that, in general, ∇·u and *s* equal zero on the examined fluid domain, except for the isolated points previously mentioned. This model requires the preservation of mass in the fluid domain Ω at all times [27]:

(5) $$\rho \int \Omega \left(\nabla \cdot u\right)dx=0$$

As the parent cell divides into two daughter cells and the process evolves, cancer cells start experiencing forces both from the surrounding extracellular matrix or non-cancerous tissue, as well as from neighboring cancer cells. The exerted force density at any time is given by the following [27]:

(6) $$f(x,t)=\int_{\Gamma }F(l,t)\delta (x-X(l,t))dl$$

where *Γ* represents a finite collection of all cells’ immersed boundaries, F(l,t) are the boundary forces, *δ* is the Dirac delta function, and X(l,t) represents the curvilinear coordinates defining the elastic cell boundaries, with l being a position on the cell boundary. The latter can be any position on the cell boundary, or a finite arclength in the initial configuration.

In addition to the extracellular and the intracellular fluids, modeling of a cancer cell requires the incorporation of several cell elements, starting with deoxyribonucleic acid (DNA) and also including the nucleus, mitochondria, cytoskeleton, cytoplasm, plasma membrane, membrane receptors, and adherens junctions.

## 3. DNA

**Figure 2:**
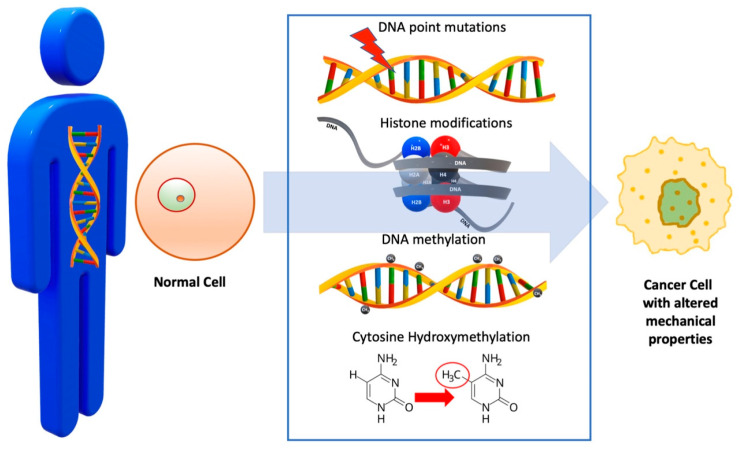
Epigenetic changes, like DNA methylation and histone modifications, affect the expression of cytokines associated with these processes, which impact carcinogenesis and tumor progression. Research has shown that DNA mechanics can affect certain DNA actions like binding and looping, packaging, and bending.

As already mentioned, normal cells acquire many mutations, which may accumulate over several years or even decades, before transformation into malignancy, a process that is influenced by a combination of physiological and metabolic pathways, as well as environmental cues. Onset changes in DNA typically occur during the initiation stage of carcinogenesis. These lesions give rise to crucial genetic mutations that disrupt normal cellular processes, leading to the activation of oncogenes or the inactivation of tumor suppressor genes, influencing both cancer development and progression (Figure 2, Table 1).

**Figure 3:**
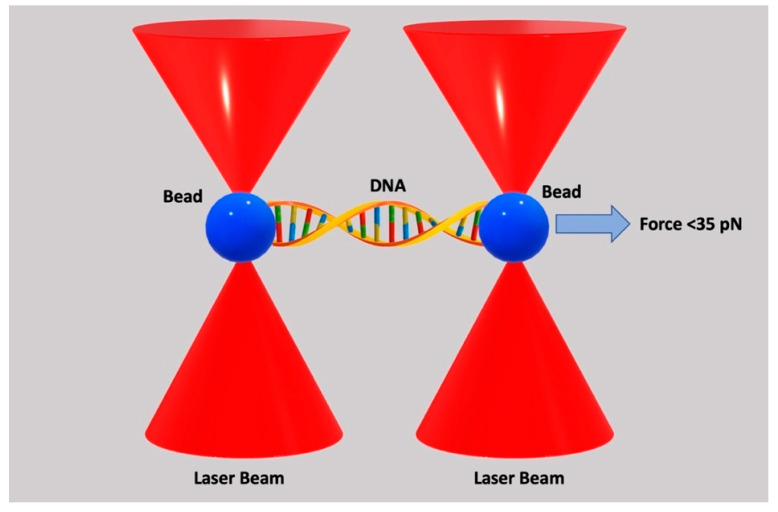
The four ends of the DNA strands are connected to optically trapped beads. When the applied stretching force is smaller than 35 pN, DNA conforms to the worm-like chain (WLC) model. When the exerted force reaches 60 pN, DNA overstretches and unpeels, and a distinct saw-tooth pattern is observed.

Among the most common epigenetic alterations observed in malignant cells are histone modifications, DNA methylation, and cytosine hydroxymethylation. These modifications play a critical role in regulating gene expression, where their deregulation can lead to abnormal cell proliferation, silencing of tumor suppressor genes, or activation of oncogenes, all contributing to cancer progression. These regulate apoptosis, autophagy, and microRNA expression [31]. Although DNA mutations are confined to the molecular level, they are either directly impacting DNA structure (e.g., histone modifications, DNA methylation, and chromosomal mutations) [32] or affecting downstream cellular biomechanics (point mutations, gain of oncogenes, etc.) [33,34]. Therefore, apart from changes to genetic pathways, DNA lesions may affect cell biomechanics, contributing to changes in the cellular space and extracellular matrix. Histone modifications, for instance, can lead to changes in chromatin compaction, altering DNA’s accessibility for critical processes such as transcription, replication, and repair. In cancer, reduced chromatin compaction can increase accessibility to transcription factors, potentially driving the expression of oncogenes or silencing tumor suppressor genes, thereby promoting uncontrolled cell growth and proliferation [35]. Methylation modulates the mechanical properties of DNA in a similar way, by indirectly affecting chromatin structure. DNA methylation involves the addition of a methyl group to cytosine residues in DNA, typically occurring at CpG dinucleotides, thereby influencing DNA accessibility to transcription factors and other regulatory proteins [36]. Cytosine hydroxymethylation instead affects DNA strand separation propensity, thus modulating the response of DNA to mechanical stress. This can impact gene expression and chromatin accessibility, highlighting the intricate relationship between hydroxymethylation and the mechanical properties of DNA [37]. Research has shown that DNA’s response to mechanical loads depends on the magnitude of the exerted force. If this is below 35 pN, the response of the DNA will be fully described by the extensible worm-like chain model, which considers DNA as an extensible and homogeneous rod [38,39,40,41] (Figure 3).

According to Gross et al. (2011), the relation between the applied force and DNA extension is given by the following [42]:

(7) $$l=L_{c}\left(1-\frac{1}{2}\sqrt{\frac{k_{B}T}{FL_{p}}}+\frac{C}{-g{\left(F\right)}^{2}+SC}F\right)$$

where l  is the extension of the DNA, $L_{c}$ is the contour length, $k_{B}$ is the Boltzmann constant, T is the absolute temperature, F is the exerted force, $L_{p}$ represents the persistence length, C is the modulus of stretching, while g(F) is the twist–stretch coupling. It should be mentioned that g(F) changes sign at forces close to 35 pN, as the DNA starts unwinding [43]. When the applied force is above 60 pN, the DNA will overstretch, following a clear saw-tooth configuration [42].

Previous research has shown that DNA mechanics can affect certain DNA actions like binding and looping, packaging, and bending. It has also been recognized that the flexibility that DNA presents is important for repair proteins to identify and bind to lesions [44,45,46,47]. It has not, however, been demonstrated until today how DNA alterations may affect DNA mechanics and the impact that this may have on cancer progression. On the contrary, it has been demonstrated in the past that epigenetic changes, like DNA methylation and histone modifications, affect the expression of cytokines associated with these processes, which impact carcinogenesis and tumor progression as cytokines have been shown to play a crucial role in angiogenesis, metastasis, and chemoresistance in gastric cancer [48].

DNA point mutations, i.e., changes in the nucleotide sequence, occur in important driver genes like BRCA2, TP53, KRAS, and BRAF [49]. Mutations in these genes not only disrupt crucial cellular processes like DNA repair and apoptosis but can also influence cellular mechanics. For example, mutations in TP53 can alter cytoskeletal organization, leading to changes in cell stiffness and deformability, both of which are critical for cancer cell migration and invasion. These mutations can also alter DNA properties in various ways, e.g., as drivers of silent mutations, where the amino acid remains the same [50], missense mutations that result in functionally different amino acids affecting protein function [51], or nonsense mutations causing the premature termination of protein synthesis and loss of function [52].

**Table 1:** DNA changes leading to alteration in its mechanical properties.

DNA Change	Mechanism	Effect on Cellular Processes	Impact on Cancer Biology
DNA Point Mutations	Point mutations in driver genes (e.g., BRCA2, TP53, KRAS) [49]	Disruption of crucial processes like DNA repair, apoptosis, and cell division, as well as alterations in cytoskeletal organization, leading to changes in cell stiffness and deformability [50]	Mutations can lead to abnormal cell proliferation, migration, and invasion and impact cancer progression and therapy [50]
Histone Modifications	Chemical modifications of histones [35]	Alters chromatin structure, affecting gene expression [35]	Modulates chromatin compaction, influencing oncogene expression, tumor suppressor silencing, and uncontrolled cell growth and proliferation [35]
DNA Methylation	Addition of methyl groups to DNA at CpG dinucleotides [36]	Affects DNA accessibility to transcription factors and other regulatory proteins [36]	Modulates gene expression, influencing oncogene activation or tumor suppressor silencing [35,36]
Cytosine Hydroxymethylation	Addition of a hydroxymethyl group to cytosine residues [37]	Affects DNA strand separation propensity, thus modulating mechanical stress response [37]	Affects chromatin accessibility, gene expression, and DNA mechanics, influencing cancer cell behavior [37]

## 4. Nucleus

The nucleus in cancer cells presents distinct differences from that observed in normal cells, with nuclear size, shape, and chromatin organization being among the key differences commonly used to diagnose malignancy. Nevertheless, not all cancer cell nuclei share the same characteristics. These depend on the type of cancer and can be used to inform diagnosis [53]. Cancer cell nuclei are usually enlarged and possess an irregular and folded shape, presenting coarse heterochromatin aggregates [54]. It has been argued that these nucleus morphological alterations in malignant cells assist in metastasis and proliferation through nuclear deformation and remodeling of the cytoskeleton.

The latter is less important, as the nucleus is much stiffer than the cytoskeleton, and its deformability is the rate-limiting step in the whole process of cell migration. Wolf et al. postulated that migration occurs through an amoeboid cell deformation, where cells squeeze through narrow spaces without relying on strong cell–matrix adhesion, and thus, the ratio of the nucleus cross-sectional area to the size of the matrix pores is important in metastasis [55]. During cancer cell migration through confined spaces, the nucleus deforms from the application of significant forces [56,57]. Previous research has demonstrated that the nucleus presents a significantly greater elastic response to mechanical loading than the cytoplasm [58,59]. However, other studies using atomic force microscopy present some contradictory findings, showing that the nucleus is stiffer than the cytoplasm [60].

These conflicting findings may result from variations in measurement techniques or differences in cell types and experimental conditions.

It has been postulated that nuclear lamina is responsible for the elastic response of the nucleus [61,62]. It consists of type V intermediate filament proteins (lamins), which create a network, having a thickness of 100 nm [17]. Recent evidence suggests that chromatin contributes also to the mechanical properties of the nucleus, and it is responsible for the elastic response of the nucleus at small deformations, while lamins A and C dictate the response at large deformations [63,64].

Following previously published research, Estabrook et al. treated the nucleus as an elastic object and provided the constitutive equations for a continuum elastic solid, to which the deformation of the nucleus should obey the following [65,66,67,68]:

(8) $$\sigma_{ij}=\frac{E}{1+v}\left(\varepsilon_{ij}+\frac{v}{1-2v}\varepsilon_{kk}\delta_{ij}\right)$$

and for isotropic elastic materials, stress is related to strain as follows [69,70]:

(9) $$\sigma_{ij}=\lambda \delta_{ij}\varepsilon_{kk}+2\mu \varepsilon_{ij}$$

where $\sigma_{ij}$ represents the stress tensor, E is the modulus of elasticity, v is Poisson’s ratio, $\varepsilon_{ij}$ is the strain tensor, $\delta_{ij}$ represents the Kronecker delta function, and λ and μ are Lamé’s first and second parameters, respectively.

The strain tensor,$\;\varepsilon_{ij},$ is given by the following:

(10) $$\varepsilon_{ij}=\frac{1}{2}\left(\frac{\vartheta u_{i}}{\vartheta x_{j}}+\frac{\vartheta u_{j}}{\vartheta x_{i}}+\frac{1}{2}\frac{\vartheta u_{k}}{\vartheta x_{i}}\frac{\vartheta u_{k}}{\vartheta x_{j}}\right)$$

where $x_{i}$ represents the position vector and $u_{i}$ is the deformation. These equations focus on the sequential deformations among different points of time, and the reference is the previous point of time. In this manner, the forces and the energy associated with the cell deformation due to passing through constricted sites are supplied, while the nucleus is assumed to be under no applied force. In contrast, the nucleus is studied as a pre-stressed elastic material due to confinement.

The relation of the stress ($\sigma_{ij})$ and strain tensors ($\varepsilon_{ij})$ to free energy (*ƒ*) is given by the following [71]:

(11) $$f=\frac{1}{2}\sigma_{ij}\varepsilon_{ij}$$

while the traction force of an elastic object is supplied by the following [71]:

(12) $$t_{i}=\sigma_{ij}n_{j}$$

where $n_{i}$ represent the components of the normal to the surface.

**Figure 4:**
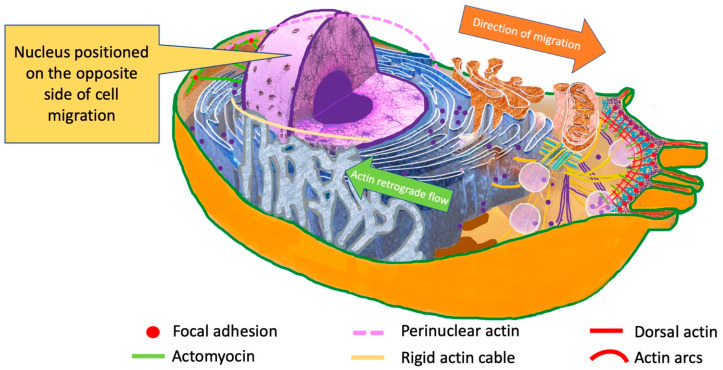
During invasion, cancer cells pass through multiple confinements, which can be smaller than the cell’s original size. During this process, the cytoskeleton and the organelles are re-arranged. The nucleus is positioned on the opposite side of that of the migration to allow the cancer cell’s passage through minute spaces. This is an essential step, as it allows for cell polarity in the migration direction, and it is propelled by an actin retrograde flow, which is facilitated by Cdc42 and myosin. Proteins SUN2 and Nesprin-2 form linear arrays in the nuclear envelope, facilitating connection with actin filaments and developing TAN lines.

During invasion, cancer cells need to pass through multiple confinements, which are sometimes smaller than the cells’ original size. Therefore, nucleus deformation during cancer cell migration is very important, as the nucleus represents the largest cell organelle [72,73,74]. In addition to deformation, the position of the nucleus seems to be significant. During the migration process, cytoskeleton and organelles are re-arranged. The nucleus is positioned on the opposite side of that of the migration [75,76,77] (Figure 4).

This is an essential step, as it allows for cell polarity in the migration direction, and it is propelled by an actin retrograde flow, which is facilitated by Cdc42 and myosin [75,77]. Actin is attached to the nuclear envelope by the Linker of Nucleoskeleton and Cytoskeleton (LINC) complex, which is the primary connection between the cytoskeleton and the nucleus. LINC complexes are composed of two protein domains, the SUN (Sad1p, UNC-84), which spans the inner nuclear membrane, and the NE nesprins (nuclear envelope spectrin-repeat proteins), which span the outer nuclear membrane [78,79,80]. The primary purpose of the LINC complexes is to transfer mechanical cues from the plasma membrane to the nucleus, resulting in biochemical reactions and activation of some sets of genes, which present mechanosensitivity [81,82].

## 5. Mitochondria

Mitochondria are tubular organelles, with a size ranging from 0.5 μm to 5 μm [17]. In healthy eukaryotic cells, these organelles control vital cellular processes, including proliferation, death, Ca^2+^ homeostasis, and metabolic adaptation. In mitochondria, important reactions also take place, including the generation of adenosine triphosphate (ATP) through oxidative phosphorylation (OXPHOS) [83]. The latter represents a complex process in which electron carriers are utilized to transfer electrons from NADH and FADH_2_ to O_2_, involving the electron transport chain (ETC). Other important processes taking place in mitochondria include the biosynthesis of amino acids, nucleotides, and lipids. They also regulate caspase activation and cell death, through the release of cytochrome c [84]. Moreover, mitochondria produce reactive oxygen species (ROS), a biochemical process which underlies oxidative damage in disease and is involved in retrograde redox signaling from the organelle to the nucleus and cytosol [85].

These normal processes are altered in cancer, where the cells are characterized by the Warburg effect in which glycolysis is upregulated, even if O_2_ is present [86]. Through this path, biosynthetic demands are supported, resulting in rapid tumor growth [87]. Other distinct differences from normal cells include elevated ROS production, which can be produced both in the tricarboxylic acid (TCA) cycle and in the ETC [88]. ROS is considered one of the most important stimuli for cancer initiation, as it promotes DNA damage, mutations, and cancer progression through matrix metalloproteinase (MMP) activation, which breaks down the extracellular matrix (ECM) [89]. Moreover, mitochondria in cancer cells present different dynamics which involve increased fission rates. It has been demonstrated that fission is a process requiring specific proteins, some of which are enzymes modifying mitochondrial membranes [90]. Fission is initiated by actin and endoplasmic reticulum (ER) tubules which delineate the area of division on the outer mitochondrial membrane (OMM) [91,92]. It has been shown that the ER releases Ca^2+^ into the mitochondrion to activate the polymerization of actin at the constriction area [93]. Involved proteins include dynamin-related protein 1 (DRP1) to the OMM by fission 1 homolog protein (FIS1), mitochondrial dynamics proteins of 49 kDa (MID49) and 51 kDa/mitochondrial elongation factor 1 (MID51/MIEF1), and the mitochondrial fission factor (MFF) [94].

The probability of mitochondria fission (*P_fission_*) is increased as their length is increased, and it is given by the following [95]:

(13) $$P_{fission}=1-exp\left(-\frac{E_{tension}+E_{bend}}{k_{B}T}\right)$$

where $E_{tension}$ is the energy from the membrane tension, $E_{bend}$ is the bending energy from DPR1 constriction, $k_{B}$ is the Boltzmann constant, and T represents the absolute temperature.

**Figure 5:**
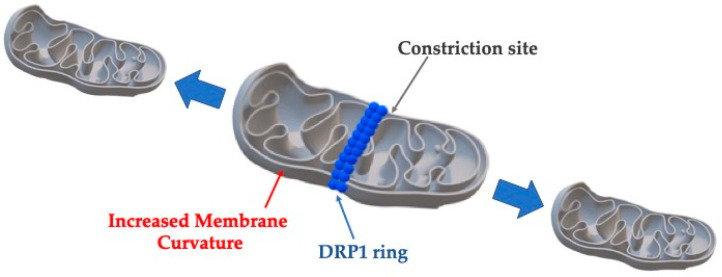
The process of mitochondrial fission requires the synergetic action of multiple proteins, changing the shape of the mitochondrion. In the middle, there is a constriction site, due to the action of DRP1, while DNM1 contributes to the increased membrane curvature, observed everywhere except the middle portion of the mitochondrion.

The total energy required for mitochondria fission is given by the following [96]:

(14) $$E_{total}=E_{tension}+E_{bending}=\sigma \cdot A+\frac{1}{2}k\int {\left(C_{1}{+C}_{2}-C_{0}\right)}^{2}dA$$

where σ represents the membrane tension, A is the area under tension, k is the bending rigidity, $C_{1}{\;and\;C}_{2}$ are principal curvatures, and $C_{0}$ is the spontaneous curvature, which is induced by DRP1 (Figure 5).

Fission affects actin dynamics through depolymerization or reorganization of the actin network at mitochondria constriction sites by relieving mechanical tension after fission is completed. Through this process, the cell’s cytoskeleton is also affected, and there is a decrease in cancer cells stiffness, making their migration through narrow passages during invasion and metastasis easier [97].

## 6. Cytoskeleton

The cytoskeleton provides structural integrity to the cell and is involved in several important cell functions, including adhesion, mechanotransduction, migration, and mitosis. The eukaryotic cytoskeleton consists of actin microfilaments (MFs) (≈7–9 nm diameter), microtubules (MTs) (≈25 nm diameter), and intermediate filaments (IFs) (≈10 nm diameter). These primary structures are interconnected and form a complex network, which is in a state of continuous flux, particularly when cellular dynamic processes take place [98]. A characteristic property of the cytoskeleton is the presence of several proteins, which form a cross-linked bundle and lattice networks, which provide elasticity to the cytoskeleton.

It has been argued in the past that the cytoskeleton contributes to tumorigenesis by inducing cell proliferation and oncogene activation [99]. Kimura et al. demonstrated that CKAP4 (cytoskeleton-associated protein 4)—which is also known as CLIMP-63—is a Dickkopf1 receptor and is associated with lung and pancreatic cancer through its role in signaling pathways that promote tumor growth [100]. Additionally, recent findings suggest the involvement of Zyxin, which is a cytoskeletal LIM-domain protein regulating actin assembly and generating traction force, in tumorigenesis, if it is non-functional [101,102].

Actin-bundling proteins and intermediate filaments are also associated with cancer progression. F-actin is organized into parallel bundles by fascin proteins, which are necessary for the development of cellular protrusions. It has been demonstrated that fascin protein suppression interferes with filopodia formation and inhibits cancer invasion and metastasis [103]. Regarding intermediate filaments, it has been argued that their decreased number can alter the cell’s shape [104]. Research has also shown that reorganization of the cytoskeleton plays an important role in metastasis and in epithelial–mesenchymal transition [105,106]. It has been demonstrated that during this transition, there is a diffusion of beta-actin distribution, as well as a reduction in the number of beta-actin fibers. Moreover, it has been observed that there is a rearrangement of intermediate filaments and a change in network proteins, from cytokeratin-rich to vimentin-rich [107].

Given the cytoskeleton’s key role in cell shape, migration, and mechanotransduction, it has become an important target for cancer therapies. Drugs that disrupt cytoskeletal components, such as actin or microtubules, can inhibit cancer cell division and migration. For example, microtubule-targeting agents like paclitaxel prevent cell division, while actin-targeting agents like cytochalasin D reduce cancer cell movement by disrupting actin polymerization [108,109]. Understanding the biomechanical properties of the cytoskeleton is critical for developing therapies that limit cancer invasion and metastasis.

From a biomechanical point of view, the cytoskeletal filaments are considered as very thin homogeneous elastic fibers. The axial stress ($\sigma_{ii})$ is given by the following [98]:

(15) $$\sigma_{ii}=E_{f}\varepsilon_{ii}$$

where $E_{f}$ represents Young’s modulus and $\varepsilon_{ii}$ is the axial strain.

The bending moment (*M*) of the fibers is given by the following:

(16) $$M=E_{f}I\left(\frac{d^{2}w}{dx^{2}}\right)$$

where *I* is the moment of inertia, *w* represents the deflection of the fiber, and *x* is the distance along the fiber axis.

The modulus of elasticity of the entire cytoskeletal network is given by the following [110]:

(17) $$E^{*}=E_{s}C_{1}{\left(\frac{\rho^{*}}{\rho_{s}}\right)}^{2}$$

where *E_s_* is the Young’s modulus of each individual cytoskeletal fiber, *C*_1_ is a constant of proportionality, $\rho^{*}$ is the average density of the whole network, and $\rho_{s}$ is the density of the fibers. The ratio $\frac{\rho^{*}}{\rho_{s}}$ represents the relative density.

Previous research on cervical cancer (HeLa) cells has demonstrated that the modulus of elasticity of the cytoskeleton is significantly decreased if cytochalasin B is administered. This cell-permeable mycotoxin is an actin polymerization inhibitor that does not affect intermediate filaments or microfilament structure [111,112]. Similarly, when cytochalasin D was used for the treatment of bladder cancer, cell deformability and reduced modulus of elasticity were observed [113,114]. These changes affect cancer cell shape changes and make migration easier through tight spaces.

## 7. Cytoplasm

The cytoplasm consists of the cytosol, which is an aqueous solution, and all cellular organelles. It is surrounded by the plasma membrane, and it represents the site where the majority of biochemical activities take place [115]. The cytoplasm comprises macromolecules, such as lipids, nucleic acids, proteins, etc., and it occupies 10–40% of the volume of the cell [116,117]. Cytoplasm density may vary as a result of cell cycle, aging, nutritional stress, or disease [118]. These density variations affect both the physical properties of the cytoplasm and the biological processes within the cell. The latter include protein–protein associations, different phase transitions, and enzymatic fluxes [119]. Research has also shown that there is a macromolecular crowding in the cytoplasm. Therefore, cytoplasm is very densely occupied by the cell’s organelles and macromolecules, and it has been shown that it behaves as a viscoelastic porous material [120,121]. This viscoelasticity allows the cytoplasm to deform under mechanical forces, which is crucial for cellular migration and shape changes, particularly in cancer cells.

As the cytoplasm constitutes a large part of the cell, its biomechanical contribution is very important and has been taken into account in many previous studies. Although, as stated earlier, the nucleus is the largest organelle and needs to be deformed during the cell’s passage through multiple confinements, the cytoplasm also needs to be compressed, as it represents a significant portion of total cellular volume.

Copos and Guy have described a two-phase flow model, trying to describe the interaction between cytoskeleton and cytoplasmic fluid dynamics [122]. In a healthy state, the cytoskeletal network represents a small volume fraction when compared to the cytoplasmic fluid phase. In cancer, a disorganization of the actin cytoskeleton is observed, leading to a further elastic/fluid ratio reduction [123]. Therefore, the elastic network can be considered as negligible, and Biot’s poroelastic standard model can be used [124]. In this model, a spherical cancer cell, with its cytoskeleton contained within the cytoplasm, is considered in a domain Ω. Independent force balance equations apply to the internal elastic stresses of the cytoskeleton and to the viscous fluid, where the solid structure is. The viscous fluid and the solid structure move with their own rate fields, and these two materials are connected through drag forces. Then, the Stokes equations apply [123]:

(18) $$\mu ∆u-\nabla p+f_{dfd}=0$$

(19) ∇·u=0

where μ represents fluid’s dynamic viscosity, u the velocity of the fluid, p the pressure, and $f_{dfd}$ is the drag force density on the viscous fluid caused by the cytoskeleton’s relative motion.

The cytoskeleton movement is given by the following:

(20) $$\frac{\partial X}{\partial t}=U=\frac{1}{\xi }F_{efd}+u$$

where $F_{efd}$ represents the elastic force density in the structure.

## 8. Plasma Membrane

**Figure 6:**
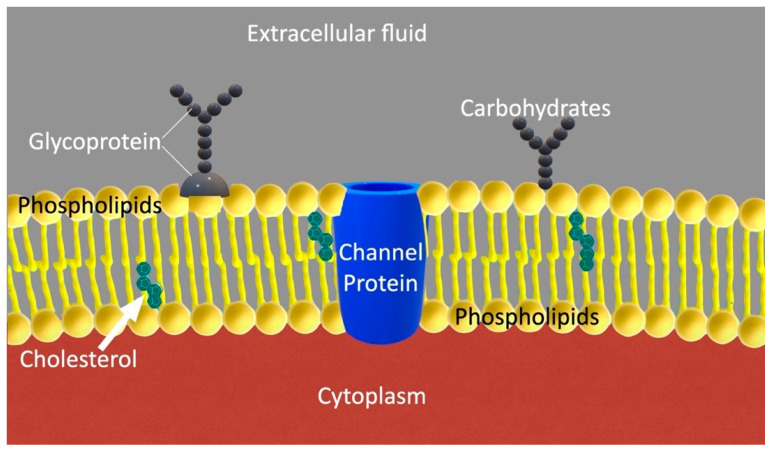
Plasma membrane, with the double phospholipid layer. In cancer cells, the phospholipid content of the plasma membrane is altered. Sphingomyelin levels are reduced, and monounsaturated and saturated phospholipids increase. These changes affect the biomechanical behavior of the membrane.

The plasma membrane is not only a barrier isolating the cell from its environment, but an organelle as well, consisting of lipids in a bilayer arrangement, including numerous specialized proteins (Figure 6), which play an active role in sensing and responding to environmental cues, a function that becomes crucial in the altered microenvironment of cancer [94]. This membrane is involved in several important cellular functions, including structural support, transportation of nutrients inside the cell, transportation of toxic substances out of the cell, biomechanical and biochemical signaling, and motion [125,126]. Furthermore, the plasma membrane is also involved in several disease processes, including cancer [127].

Published evidence has shown that the phospholipid content of plasma membrane in cancer is altered, which affects membrane fluidity and stiffness. Specifically, it has been demonstrated that sphingomyelin levels are reduced, while monounsaturated and saturated phospholipids are higher in cancer cells, when compared to normal cells [128]. These changes are also associated with changes in the biomechanical behavior of plasma membranes.

Previous research has demonstrated that Fourier analysis can be used to detect alterations in the bending rigidity between normal and malignant cells [129,130,131]. It has been advocated in the past that plasma membrane plays a significant role in metastasis, as in cancer cells the cytoskeleton decouples locally from the membrane, affecting its rigidity [132,133]. Other researchers have speculated that there is a correlation among phenotype, cell signaling, cell membrane alterations, and cellular motion. The epithelial–mesenchymal transition (EMT) summarizes these ideas [57,90]. Cellular changes in elasticity have been studied with a microfluidic optical stretcher [134].

Giant plasma membrane vesicles (GPMVs) have been used in the past to determine the rigidity of plasma membranes, without the contribution of the cytoskeleton, which, in cancer, decouples from the membrane and does not contribute to its mechanical properties. GPMVs are produced from cells by employing the vesiculation process during which the plasma membrane disconnects from the cytoskeleton. GPMVs are basically spheres of 1 to 100 μm, consisting of two layers [135]. The rigidity (κ) of the plasma membrane in GPMVs is given by the following [136]:

(21) $$\kappa =\frac{\langle {\left|u_{lm}\right|}^{2}\rangle (l+2)(l-1)\left[l(l+1)+\bar{\sigma }\right]}{k_{B}T}$$

(22) $$\bar{\sigma }=\sigma_{eff}\frac{R^{2}}{\kappa }$$

where $u_{lm}$ represents the mean square amplitudes of the spherical harmonics, l is the azimuthal length, $\sigma_{eff}$ is the effective tension, R is the average radius, *k_B_* is the Boltzmann constant, and *T* is the temperature.

According to Goetz et al., in the absence of numerical data, the plasma membrane bending stiffness can be calculated by the following [136]:

(23) $$\kappa =\frac{K_{a}h^{2}}{\gamma }$$

where $K_{a}$ is the modulus of stretching elasticity, h is the thickness of the plasma membrane, and γ represents a numerical constant, which, according to molecular dynamics simulations and in agreement with classical elasticity theory, leads to γ ≅ 48 [137].

Experimental results suggest that the mechanical properties of plasma membranes are greatly affected by their lipid components. Gracia et al. argued that cholesterol influences the mechanical properties of the membrane and its bending stiffness, by modifying the acyl chain order and the interfacial membrane region [136].

## 9. Membrane Receptors—Adherens Junctions

**Figure 7:**
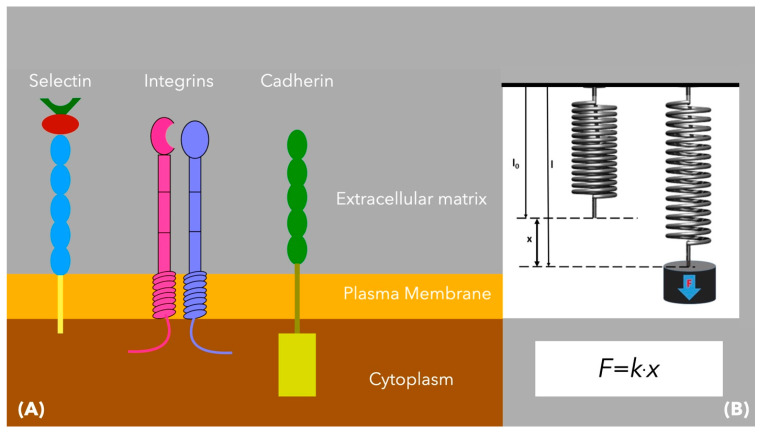
(**A**) The main adhesion proteins providing connection between cancer cells or between cancer cells and normal cells are the integrins, cadherins, and selectins. (**B**) In the model of Rejniak [19,27], these proteins are modeled as linear springs, satisfying Hooke’s law.

As each cancer cell is in contact with adjacent malignant or healthy cells, its plasma membrane has been modeled as a group of points [19,27]. These points refer to transmembrane adhesion proteins, mediating cell-to-cell interactions and mechanical communication between cells. The most important of these proteins include integrins, cadherins, and selectins. These have been modeled as linear springs, satisfying Hooke’s law (Figure 7).

**Figure 8:**
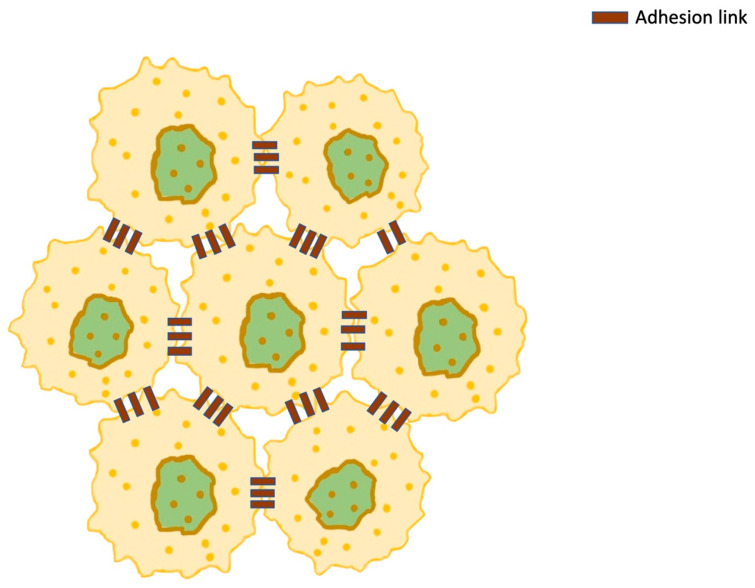
A cluster of cancer cells interconnected with transmembrane adhesion proteins (adhesion links), which exert adhesive forces (*F_adh_*). The forces can also be repulsive, if the cells approach too much. Then, $F_{rep}$ will take over in order to deter cell interpenetration, and when the distance becomes sufficient, $F_{adh}$ will prevent cell detachment.

Therefore, the force applied to every point on the plasma membrane is as follows [19,27] (Figure 8):

(24) $$F_{adh}\left(l,t\right)=F_{adh}\frac{\left\|P\left(k,t\right)-P\left(l,t\right)\right\|-L_{adh}}{\left\|P\left(k,t\right)-P\left(l,t\right)\right\|}\left(P\left(k,t\right)-P\left(l,t\right)\right)$$

where $P\left(l,t\right)$ is the point to which the force is applied by a neighboring point $P\left(k,t\right)$, and $L_{adh}$ represents a constant resting length of the membrane protein, which is modeled as a spring. These points have receptors, which are used for communication purposes and enable adhesion to adjacent cells. However, the forces between neighboring cells can also be repulsive if the cells approach too much. Then, $F_{rep}$ will take over in order to deter cell interpenetration, and when the distance becomes sufficient, $F_{adh}$ will prevent cell detachment. In between these two distances, there is a distance of equilibrium, at which $F_{rep}$ and $F_{adh}$ vanish [138]. Different approaches have been used to explain the bonds between neighboring cells. Van Liedekerke et al. assumed that permanent adhesive bonds exist, while Sanderius and Newman adopted a model in which a Morse potential exists on the connection points of the membranes of the cells [139,140].

Previous research has shown that cell-to-cell communication is very important in cancer expansion and disease progression. This process starts in the tumor microenvironment ${\;\Theta }_{\Gamma }$, and then progresses at more distant sites [141]:

(25) $$\Theta_{\Gamma }={⋃}_{x_{k}\in \Gamma }\;\left\{x:∥x-X_{k}∥<\varepsilon \right\}$$

where $x_{k}\in \Gamma$ is the cell boundary, and ε is the radius of the microenvironment.

Cellular communication in cancer happens with direct intercellular communication, involving the exchange of different molecules, ions, and electrical impulses between cells through *gap junction channels* formed by connexons [142]. Other ways of intercellular communication include ligand (L)-receptor (R) interactions [142]. It has been recognized that L-R communication between two adjacent cells may present some restrictions, due to paired L-R expression intensity/specificity. Zhang et al. presented the communication score (*S_k_*) between two neighboring cells [13]:

(26) $$S_{k}={\left\|\underset{{LR}_{k}}{\to }\right\|}_{2}\times {TF}_{k}$$

where $\underset{{LR}_{k}}{\to }$ is a two-dimensional vector associated with the mean expression value of ligand (L) in the first cancer cell and with that of the receptor (R) in the second cancer cell, while *TF_k_* represents the activity score of the transcription factors (TFs) downstream of the interaction (*k*) between L and R.

The continuous biochemical–biomechanical interactions taking place within the cancer cell have an effect on the organelles, altering their physical and mechanical properties, affecting normal biological processes and assisting in cancer progression and metastasis (Table 2).

## 10. Discussion

Biochemical pathways are crucial in tumorigenesis and disease progression. Nevertheless, research has demonstrated that mechanical signals have an equally important role, as they contribute to cell differentiation and fate. Understanding the mechanical behavior of cancer cells at the organelle level is essential for offering insight into how they invade, survive, and adapt to challenging microenvironments. Mathematical models in this review provide a framework for quantifying these biomechanical properties and linking them to cellular function and pathology.

The mathematical representation in Equations (1) and (2) models the cell as a deformable elastic entity, where mitotic spindle forces are applied at opposed points (P1, P2) and describes the concept of a minimum threshold distance $L_{div}^{min}$, which is critical for mitosis. The model’s assumptions, while simplifying cytoskeletal and membrane complexity, provide key principles of force-driven mitosis and spatial cell constraints. Minc et al. also demonstrated that spatial confinement can influence spindle alignment and the symmetry of cell division [143]. Additionally, the Navier–Stokes equations presented in this review (Equations (3) and (4)) treat cancer cells as elastic spheroids embedded in an incompressible viscous medium. This model offers understanding of how extracellular and intracellular fluid dynamics influence tumor growth, emphasizing the relationship between mechanical forces and the extracellular matrix (ECM) [27]. The assumption of increased viscosity which is adopted—instead of modeling the cytoskeleton explicitly—is a reasonable compromise for computational feasibility. However, it should be noted that greater accuracy could be obtained if fiber networks are incorporated into future models. Similar models have precedent in studies like those by Drasdo and Hoehme [144].

The literature suggests that nuclear deformation is a decisive factor during the transmigration of cells through narrow ECM pores [55,63,145]. The mathematical models presented in Equations (8)–(11) treat the nucleus as an elastic solid using continuum mechanics principles. Many studies have demonstrated that the nuclear envelope is significantly stiffer than the cytoplasm [55,59,64,146]. Clinically, this approach turns nuclear stiffness into a biomarker for metastatic aggressiveness and into a therapeutic target, for instance, via the modulation of lamin A/C expression, as has been described in previous studies [147].

Models describing DNA as a worm-like chain (WLC)—as in the studies of Bustamante et al. and that of Marko and Siggia—have been extended to incorporate torsional stress and chromatin packing, both of which affect transcription, replication, and repair [148,149]. The bending and looping presented in these models contribute to understanding how external mechanical forces on DNA can activate oncogenic pathways. Equations (15) and (16) model cytoskeletal elements, i.e., actin and microtubules, as linearly elastic rods, capturing axial stress and bending moments. These formulations are supported by experimental research data from atomic force microscopy (AFM) and optical tweezers, which has demonstrated that cytoskeletal stiffness correlates with the tumor’s metastatic potential [150,151]. The relationship of the effective modulus of the cytoskeletal network to fiber density and arrangement, in Equation (17), has been confirmed in several studies, where changes in actin density or architecture lead to measurable reductions in cell stiffness [152]. Nevertheless, the model of Storm et al. incorporates nonlinear elasticity and strain stiffening, suggesting further investigation [153].

Modeling the cytoplasm as a poroelastic material, using Biot’s framework, provides a method for representing the interaction between cytoplasmic fluid and the elastic cytoskeletal matrix. This approach is supported by the work of Moeendarbary et al., which demonstrated that poroelastic stress relaxation occurs on timescales relevant to cellular deformation [120]. Equations (18) and (19) further incorporate Stokes flow principles, describing viscous flow under cytoskeletal drag. This is consistent with findings from Petrie et al., who observed that under shear stress, cytoplasmic flow modulates organelle positioning and nuclear deformation, directly affecting gene expression and migration capacity [154]. Equation (20), which couples elastic force density with cytoskeletal displacement, captures how force generation and cytoplasmic drag affect movement. This is supported by the work of Charras and Sahai, who have shown that cytoskeletal remodeling, when decoupled from cytoplasmic viscosity, through actomyosin inhibition, leads to diminished motility and altered intracellular stress distribution [155].

The mechanical behavior of cancer cells is shaped by the dynamic interaction among the nucleus, mitochondria, and cytoskeleton, and these systems interact through force transmission and biochemical signaling pathways. Understanding these interdependencies is essential for advancing cancer modeling and developing therapies that target the mechanical vulnerabilities of cancer cells during invasion and metastasis.

Future research in this field should focus on developing multiscale mathematical models that combine intracellular mechanics with tissue-level properties, taking into consideration tumor heterogeneity, ECM stiffness gradients, and microvascular dynamics. These approaches may shed light on this area and establish connections between molecular alterations, such as mutations of the KRAS oncogene and TP53 suppressor gene, with biomechanical behaviors. This approach may prove useful in helping us understand tumor evolution and consequently assist in developing targeted cancer therapy.

## 11. Conclusions

This review has focused on the mathematical relationships governing cancer mechanobiology and investigated how the altered mechanical properties of a cancer cell may affect malignant progression. DNA mutations, which can be in the form of deletions, insertions, substitutions, rearrangements, and genomic amplifications, play a significant role in cancer development and in altering the mechanical properties and biomechanical behavior of every cell organelle. A continuous biochemical–biomechanical interaction exists, ultimately affecting cell behavior. The mechanobiochemical homeostatic equilibrium is disrupted, and as a result, the mechanical properties of the cells and the tissues involved change. The extent to which these changes may affect cancer progression and response to therapeutic interventions is not yet known and needs to be further explored with in vitro and in silico studies. Despite the valuable insights gained, cancer mechanobiological modeling remains a relatively new research area, characterized by limited knowledge, insufficient data, and limited research, all of which complicate comparative analysis. Therefore, future efforts should prioritize developing standardized frameworks and analytical methodology to facilitate comparisons and advance our understanding of carcinogenesis and tumor progression.

## Figures and Tables

**Table 2:** Contribution of each cell’s organelle to cancer initiation and evolution.

Organelle	Effect	Mechanism	Molecules Involved
Nucleus	Assists in metastasis and proliferation [55] Guides the cell migration direction [75,76]	Nuclear deformation and remodeling of the cytoskeleton [56,57] Nucleus is positioned on the opposite side of that of the migration [75,76]	Chromatin (small deformations) and lamins A and C (large deformations) [63,64] Actin retrograde flow, which is facilitated by Cdc42 and myosin [75]
Cytoskeleton	Adhesion, mechanotransduction, migration and mitosis, probably induces cell proliferation and oncogene activation [84]	Development of cellular protrusions [88] Generation of traction force [86,87] Rearrangement of intermediate filaments [92]	F-actin is organized into parallel bundles [88] Actin assembly regulation by Zyxix (a cytoskeletal LIM-domain protein) [86,87] Diffusion of beta-actin distribution and reduction in the number of beta-actin fibers [90,91]
Cytoplasm	Density variations affect both the physical properties of cytoplasm and the biological processes [103]	Cytoplasm compression and disorganization of the actin cytoskeleton lead to reduction in the elastic/fluid ratio [107]	Protein–protein associations, enzymatic fluxes [103]
Plasma membrane	Involved in the cancer process [111]	Phospholipid content of plasma membrane in cancer is altered [112] Cytoskeleton decouples locally from the membrane, affecting its rigidity [116,117]	Sphingomyelin levels are reduced, while monounsaturated and saturated phospholipids are higher in cancer cells [112] Disconnection of GPMV plasma membrane from the cytoskeleton [119]
Receptors/adherens	Cancer expansion and disease progress [125]	Malignant cell-to-cell communication and adhesion [125]	Gap junction channels formed by connexons [126] Ligand (L)–receptor (R) interactions [127]

## Abbreviations

The following abbreviations are used in this manuscript:

COSMIC	Catalog of Somatic Mutations in Cancer
ECM	Extracellular Matrix
VEGF	Vascular Endothelial Growth Factor
DNA	Deoxyribonucleic Acid
BRCA2	Breast Cancer Gene 2
TP53	Gene Providing Instruction for Tumor Protein p53
KRAS	Gene Providing Instructions for Making a Protein Called K-Ras
ATP	Adenosine Triphosphate
ROS	Reactive Oxygen Species
OXPHOS	Oxidative Phosporylation
NAD	Nicotinamide Adenine Dinucleotide
FAD	Flavin Adenine Dinucleotide
ETC	Electron Transport Chain
TCA	Tricarboxylic Acid
DPR1	Dynamin-Related Protein
OMM	Outer Mitochondrial Membrane
FIS1	Fission 1 Homolog Protein
MID	Mitochondrial Dynamics Proteins
MIEF	Mitochondrial Elongation Factor
MFF	Mitochondrial Fission Factor
ER	Endoplasmic Reticulum
MMP	Matrix Metalloproteinase
EMT	Epithelial–Mesenchymal Transition
GPMV	Giant Plasma Membrane Vesicle

## References

[1] Kleeff J., Korc M., Apte M., La Vecchia C., Johnson C.D., Biankin A.V., Neale R.E., Tempero M., Tuveson D.A., Hruban R.H. (2016). Pancreatic cancer. Nat. Rev. Dis. Primers.

[2] Richters A., Aben K.K.H., Kiemeney L.A.L.M. (2020). The global burden of urinary bladder cancer: An update. World J. Urol..

[3] Pedraza-Fariña L.G. (2006). Mechanisms of oncogenic cooperation in cancer initiation and metastasis. Yale J. Biol. Med..

[4] Forbes S.A., Beare D., Gunasekaran P., Leung K., Bindal N., Boutselakis H., Ding M., Bamford S., Cole C., Ward S. (2015). COSMIC: Exploring the world’s knowledge of somatic mutations in human cancer. Nucleic Acids Res..

[5] Futreal P.A., Coin L., Marshall M., Down T., Hubbard T., Wooster R., Rahman N., Stratton M.R. (2004). A census of human cancer genes. Nat. Rev. Cancer.

[6] Lawrence M.S., Stojanov P., Mermel C.H., Robinson J.T., Garraway L.A., Golub T.R., Meyerson M., Gabriel S.B., Lander E.S., Getz G. (2014). Discovery and saturation analysis of cancer genes across 21 tumour types. Nature.

[7] Perou C.M., Jeffrey S.S., van de Rijn M., Rees C.A., Eisen M.B., Ross D.T., Pergamenschikov A., Williams C.F., Zhu S.X., Lee J.C. (1999). Distinctive gene expression patterns in human mammary epithelial cells and breast cancers. Proc. Natl. Acad. Sci. USA.

[8] Perou C.M., Sørlie T., Eisen M.B., van de Rijn M., Jeffrey S.S., Rees C.A., Pollack J.R., Ross D.T., Johnsen H., Akslen L.A. (2000). Molecular portraits of human breast tumours. Nature.

[9] Garber M.E., Troyanskaya O.G., Schluens K., Petersen S., Thaesler Z., Pacyna-Gengelbach M., van de Rijn M., Rosen G.D., Perou C.M., Whyte R.I. (2001). Diversity of gene expression in adenocarcinoma of the lung. Proc. Natl. Acad. Sci. USA.

[10] McBeath R., Pirone D.M., Nelson C.M., Bhadriraju K., Chen C.S. (2004). Cell shape, cytoskeletal tension, and RhoA regulate stem cell lineage commitment. Dev. Cell.

[11] Engler A.J., Sen S., Sweeney H.L., Discher D.E. (2006). Matrix elasticity directs stem cell lineage specification. Cell.

[12] Kahn J., Shwartz Y., Blitz E., Krief S., Sharir A., Breitel D.A., Rattenbach R., Relaix F., Maire P., Rountree R.B. (2009). Muscle contraction is necessary to maintain joint progenitor cell fate. Dev. Cell.

[13] Zhang H., Landmann F., Zahreddine H., Rodriguez D., Koch M., Labouesse M. (2011). A tension-induced mechanotransduction pathway promotes epithelial morphogenesis. Nature.

[14] Monier B., Gettings M., Gay G., Mangeat T., Schott S., Guarner A., Suzanne M. (2015). Apico-basal forces exerted by apoptotic cells drive epithelium folding. Nature.

[15] Maître J.L., Turlier H., Illukkumbura R., Eismann B., Niwayama R., Nédélec F., Hiiragi T. (2016). Asymmetric division of contractile domains couples cell positioning and fate specification. Nature.

[16] Broders-Bondon F., Nguyen Ho-Bouldoires T.H., Fernandez-Sanchez M.E., Farge E. (2018). Mechanotransduction in tumor progression: The dark side of the force. J. Cell Biol..

[17] Alberts B., Bray D., Lewis J., Raff M., Roberts K., Watson J.D. (2002). Molecular Biology of the Cell.

[18] Zhang S., Xiao X., Yi Y., Wang X., Zhu L., Shen Y., Lin D., Wu C. (2024). Tumor initiation and early tumorigenesis: Molecular mechanisms and interventional targets. Signal Transduct. Target. Ther..

[19] Rejniak K.A. (2005). A single-cell approach in modeling the dynamics of tumor microregions. Math. Biosci. Eng..

[20] Liberti M.V., Locasale J.W. (2016). The Warburg Effect: How Does it Benefit Cancer Cells?. Trends Biochem. Sci..

[21] Qin X., Li T., Li S., Yang H., Wu C., Zheng C., You F., Liu Y. (2020). The tumor biochemical and biophysical microenvironments synergistically contribute to cancer cell malignancy. Cell Mol. Immunol..

[22] Hanahan D., Weinberg R.A. (2011). Hallmarks of cancer: The next generation. Cell.

[23] Wicks E.E., Semenza G.L. (2022). Hypoxia-inducible factors: Cancer progression and clinical translation. J. Clin. Investig..

[24] Xin Y., Li K., Huang M., Liang C., Siemann D., Wu L., Tan Y., Tang X. (2023). Biophysics in tumor growth and progression: From single mechano-sensitive molecules to mechanomedicine. Oncogene.

[25] De Felice F., Malerba S., Nardone V., Salvestrini V., Calomino N., Testini M., Boccardi V., Desideri I., Gentili C., De Luca R. (2025). Progress and challenges in integrating nutritional care into oncology practice: Results from a national survey on behalf of the NutriOnc research group. Nutrients.

[26] Jain R.K. (2014). Antiangiogenesis strategies revisited: From starving tumors to alleviating hypoxia. Cancer Cell.

[27] Rejniak K.A. (2007). An immersed boundary framework for modelling the growth of individual cells: An application to the early tumour development. J. Theor. Biol..

[28] Rejniak K.A., Dillon R.H. (2007). A single cell-based model of the ductal tumour microarchitecture. Comput. Math. Methods Med..

[29] Peskin C.S. (1977). Numerical analysis of blood flow in the heart. J. Comput. Phys..

[30] Batchelor G.K. (2007). An Introduction to Fluid Dynamics.

[31] Sarkar S., Horn G., Moulton K., Oza A., Byler S., Kokolus S., Longacre M. (2013). Cancer development, progression, and therapy: An epigenetic overview. Int. J. Mol. Sci..

[32] Herceg Z., Hainaut P. (2007). Genetic and epigenetic alterations as biomarkers for cancer detection, diagnosis and prognosis. Mol. Oncol..

[33] Bohmann D., Bos T.J., Admon A., Nishimura T., Vogt P.K., Tjian R. (1987). Human proto-oncogene c-jun encodes a DNA binding protein with structural and functional properties of transcription factor AP-1. Science.

[34] Wang D., Qian X., Sanchez-Solana B., Tripathi B.K., Durkin M.E., Lowy D.R. (2020). Cancer-associated point mutations in the DLC1 tumor suppressor and other Rho-GAPs occur frequently and are associated with decreased function. Cancer Res..

[35] Jurga S., Barciszewski J. (2019). The DNA, RNA, and Histone Methylomes.

[36] Szyf M. (1994). DNA methylation properties: Consequences for pharmacology. Trends Pharmacol. Sci..

[37] Severin P.M.D., Zou X., Schulten K., Gaub H.E. (2013). Effects of cytosine hydroxymethylation on DNA strand separation. Biophys. J..

[38] Marko J.F., Siggia E.D. (1994). Fluctuations and supercoiling of DNA. Science.

[39] Odjik T. (1995). Stiff chains and filaments under tension. Macromolecules.

[40] Smith S.B., Cui Y., Bustamante C. (1996). Overstretching B-DNA: The elastic response of individual double-stranded and single-stranded DNA molecules. Science.

[41] Wang M.D., Yin H., Landick R., Gelles J., Block S.M. (1997). Stretching DNA with optical tweezers. Biophys. J..

[42] Gross P., Laurens N., Oddershede L., Bockelmann U., Peterman E.J.G., Wuite G.J.L. (2011). Quantifying how DNA stretches, melts and changes twist under tension. Nat. Phys..

[43] Gore J., Bryant Z., Nöllmann M., Le M.U., Cozzarelli N.R., Bustamante C. (2006). DNA overwinds when stretched. Nature.

[44] Garcia H.G., Grayson P., Han L., Inamdar M., Kondev J., Nelson P.C., Phillips R., Widom J., Wiggins P.A. (2007). Biological consequences of tightly bent DNA: The other life of a macromolecular celebrity. Biopolymers.

[45] Huo Y.X., Zhang Y.T., Xiao Y., Zhang X., Buck M., Kolb A., Wang Y.P. (2009). IHF-binding sites inhibit DNA loop formation and transcription initiation. Nucleic Acids Res..

[46] Sharma M., Predeus A.V., Mukherjee S., Feig M. (2013). DNA bending propensity in the presence of base mismatches: Implications for DNA repair. J. Phys. Chem. B.

[47] Afek A., Shi H., Rangadurai A., Sahay H., Senitzki A., Xhani S., Fang M., Salinas R., Mielko Z., Pufall M.A. (2020). DNA mismatches reveal conformational penalties in protein-DNA recognition. Nature.

[48] Reyes M.E., Pulgar V., Vivallo C., Ili C.G., Mora-Lagos B., Brebi P. (2024). Epigenetic modulation of cytokine expression in gastric cancer: Influence on angiogenesis, metastasis and chemoresistance. Front. Immunol..

[49] Ondraskova K., Sebuyoya R., Moranova L., Holcakova J., Vonka P., Hrstka R., Bartosik M. (2023). Electrochemical biosensors for analysis of DNA point mutations in cancer research. Anal. Bioanal. Chem..

[50] Zheng S., Kim H., Verhaak R.G.W. (2014). Silent mutations make some noise. Cell.

[51] Tan K.P., Kanitkar T.R., Kwoh C.K., Madhusudhan M.S. (2021). Packpred: Predicting the functional effect of missense mutations. Front. Mol. Biosci..

[52] Wittenstein A., Caspi M., Rippin I., Elroy-Stein O., Eldar-Finkelman H., Thoms S., Rosin-Arbesfeld R. (2024). Nonsense mutation suppression is enhanced by targeting different stages of the protein synthesis process. PLoS Biol..

[53] Kim D.H., Hah J., Wirtz D., Dong C., Zahir N., Konstantopoulos K. (2018). Mechanics of the cell nucleus. Biomechanics in Oncology.

[54] Zink D., Fischer A.H., Nickerson J.A. (2004). Nuclear structure in cancer cells. Nat. Rev. Cancer.

[55] Wolf K., Te Lindert M., Krause M., Alexander S., Te Riet J., Willis A.L., Hoffman R.M., Figdor C.G., Weiss S.J., Friedl P. (2013). Physical limits of cell migration: Control by ECM space and nuclear deformation and tuning by proteolysis and traction force. J. Cell Biol..

[56] Yamazaki D., Kurisu S., Takenawa T. (2005). Regulation of cancer cell motility through actin reorganization. Cancer Sci..

[57] Chaffer C.L., Weinberg R.A. (2011). A perspective on cancer cell metastasis. Science.

[58] Guilak F., Tedrow J.R., Burgkart R. (2000). Viscoelastic properties of the cell nucleus. Biochem. Biophys. Res. Commun..

[59] Rowat A.C., Lammerding J., Ipsen J.H. (2006). Mechanical properties of the cell nucleus and the effect of emerin deficiency. Biophys. J..

[60] Mathur A.B., Truskey G.A., Reichert W.M. (2000). Atomic force and total internal reflection fluorescence microscopy for the study of force transmission in endothelial cells. Biophys. J..

[61] Lammerding J., Fong L.G., Ji J.Y., Reue K., Stewart C.L., Young S.G., Lee R.T. (2006). Lamins A and C but not lamin B1 regulate nuclear mechanics. J. Biol. Chem..

[62] Harada T., Swift J., Irianto J., Shin J.W., Spinler K.R., Athirasala A., Diegmiller R., Dingal P.C., Ivanovska I.L., Discher D.E. (2014). Nuclear lamin stiffness is a barrier to 3D migration, but softness can limit survival. J. Cell Biol..

[63] Stephens A.D., Banigan E.J., Adam S.A., Goldman R.D., Marko J.F. (2017). Chromatin and lamin A determine two different mechanical response regimes of the cell nucleus. Mol. Biol. Cell.

[64] Stephens A.D., Banigan E.J., Marko J.F. (2019). Chromatin’s physical properties shape the nucleus and its functions. Curr. Opin. Cell Biol..

[65] Caille N., Thoumine O., Tardy Y., Meister J.J. (2002). Contribution of the nucleus to the mechanical properties of endothelial cells. J. Biomech..

[66] La Berre M., Liu Y.J., Hu J., Maiuri P., Benichou O., Voituriez R., Chen Y., Piel M. (2013). Geometric friction directs cell migration. Phys. Rev. Lett..

[67] Giverso C., Grillo A., Preziosi L. (2014). Influence of nucleus deformability on cell entry into cylindrical structures. Biomech. Model. Mechanobiol..

[68] Estabrook I.D., Thiam H.R., Piel M., Hawkins R.J. (2021). Calculation of the force field required for nucleus deformation during cell migration through constrictions. PLoS Comput. Biol..

[69] Sokolnikoff I.S. (1983). Mathematical Theory of Elasticity.

[70] Fung Y.C. (1993). Biomechanics: Mechanical Properties of Living Tissues.

[71] Landau L.D., Pitaevskii L.P., Lifshitz E.M., Kosevich A.M. (1986). Theory of Elasticity.

[72] Wolf K., Müller R., Borgmann S., Bröcker E.B., Friedl P. (2003). Amoeboid shape change and contact guidance: T-lymphocyte crawling through fibrillar collagen is independent of matrix remodeling by MMPs and other proteases. Blood.

[73] Davidson P.M., Denais C., Bakshi M.C., Lammerding J. (2014). Nuclear deformability constitutes a rate-limiting step during cell migration in 3-D environments. Cell Mol. Bioeng..

[74] Wu Y., Pegoraro A.F., Weitz D.A., Janmey P., Sun S.X. (2022). The correlation between cell and nucleus size is explained by an eukaryotic cell growth model. PLoS Comput. Biol..

[75] Gomes E.R., Jani S., Gundersen G.G. (2005). Nuclear movement regulated by Cdc42, MRCK, myosin, and actin flow establishes MTOC polarization in migrating cells. Cell.

[76] Tsai L.H., Gleeson J.G. (2005). Nucleokinesis in neuronal migration. Neuron.

[77] Calero-Cuenca F.J., Janota C.S., Gomes E.R. (2018). Dealing with the nucleus during cell migration. Curr. Opin. Cell Biol..

[78] Zhang Q., Skepper J.N., Yang F., Davies J.D., Hegyi L., Roberts R.G., Weissberg P.L., Ellis J.A., Shanahan C.M. (2001). Nesprins: A novel family of spectrin-repeat-containing proteins that localize to the nuclear membrane in multiple tissues. J. Cell Sci..

[79] Lombardi M.L., Jaalouk D.E., Shanahan C.M., Burke B., Roux K.J., Lammerding J. (2011). The interaction between nesprins and sun proteins at the nuclear envelope is critical for force transmission between the nucleus and cytoskeleton. J. Biol. Chem..

[80] Meier I. (2016). LINCing the eukaryotic tree of life—Towards a broad evolutionary comparison of nucleocytoplasmic bridging complexes. J. Cell Sci..

[81] Maniotis A.J., Chen C.S., Ingber D.E. (1997). Demonstration of mechanical connections between integrins, cytoskeletal filaments and nucleoplasm that stabilize nuclear structure. Proc. Natl. Acad. Sci. USA.

[82] Wang N., Tytell J.D., Ingber D.E. (2009). Mechanotransduction at a distance: Mechanically coupling the extracellular matrix with the nucleus. Nat. Rev. Mol. Cell Biol..

[83] Harris D.A., Das A.M. (1991). Control of mitochondrial ATP synthesis in the heart. Biochem. J..

[84] Liu X., Kim C.N., Yang J., Jemmerson R., Wang X. (1996). Induction of apoptotic program in cell-free extracts: Requirement for dATP and cytochrome c. Cell.

[85] Murphy M.P. (2009). How mitochondria produce reactive oxygen species. Biochem. J..

[86] Warburg O., Wind F., Negelein E. (1927). The metabolism of tumors in the body. J. Gen. Physiol..

[87] Hitosugi T., Kang S., Vander Heiden M.G., Chung T.W., Elf S., Lythgoe K., Dong S., Lonial S., Wang X., Chen G.Z. (2009). Tyrosine phosphorylation inhibits PKM2 to promote the Warburg effect and tumor growth. Sci. Signal..

[88] Galvan D.L., Badal S.S., Long J., Chang B.H., Schumacker P.T., Overbeek P.A., Danesh F.R. (2017). Real-time in vivo mitochondrial redox assessment confirms enhanced mitochondrial reactive oxygen species in diabetic nephropathy. Kidney Int..

[89] Kumari S., Badana A.K., Murali M.G., Shailender G., Malla R. (2018). Reactive oxygen species: A key constituent in cancer survival. Biomark. Insights.

[90] Scott I., Youle R.J. (2010). Mitochondrial fission and fusion. Essays Biochem..

[91] Friedman J.R., Lackner L.L., West M., DiBenedetto J.R., Nunnari J., Voeltz G.K. (2011). ER tubules mark sites of mitochondrial division. Science.

[92] Manor U., Bartholomew S., Golani G., Christenson E., Kozlov M., Higgs H., Spudich J., Lippincott-Schwartz J. (2015). A mitochondria-anchored isoform of the actin-nucleating spire protein regulates mitochondrial division. eLife.

[93] Steffen J., Koehler C.M. (2018). ER-mitochondria contacts: Actin dynamics at the ER control mitochondrial fission via calcium release. J. Cell Biol..

[94] Sebastián D., Palacín M., Zorzano A. (2017). Mitochondrial dynamics: Coupling mitochondrial fitness with healthy aging. Trends Mol. Med..

[95] Mahecic D., Carlini L., Kleele T., Colom A., Goujon A., Matile S., Roux A., Manley S. (2021). Mitochondrial membrane tension governs fission. Cell Rep..

[96] Leinheiser A.K., Mitchell C.C., Rooke E., Strack S., Grueter C.E. (2024). A dynamical systems model for the total fission rate in Drp1-dependent mitochondrial fission. PLoS Comput. Biol..

[97] Hatch A.L., Gurel P.S., Higgs H.N. (2014). Novel roles for actin in mitochondrial fission. J. Cell Sci..

[98] Mofrad M.R.K., Kamm R.D. (2010). Cytoskeletal Mechanics: Models and Measurements.

[99] Gaspar P., Holder M.V., Aerne B.L., Janody F., Tapon N. (2015). Zyxin antagonizes the FERM protein expanded to couple F-actin and Yorkie-dependent organ growth. Curr. Biol..

[100] Kimura H., Fumoto K., Shojima K., Nojima S., Osugi Y., Tomihara H., Eguchi H., Shintani Y., Endo H., Inoue M. (2016). CKAP4 is a Dickkopf1 receptor and is involved in tumor progression. J. Clin. Investig..

[101] Vouyovitch C.M., Perry J.K., Liu D.X., Bezin L., Vilain E., Diaz J.J., Lobie P.E., Mertani H.C. (2016). WNT4 mediates the autocrine effects of growth hormone in mammary carcinoma cells. Endocr. Relat. Cancer.

[102] Parshina E.A., Eroshkin F.M., Orlov E.E., Gyoeva F.K., Shokhina A.G., Staroverov D.B., Belousov V.V., Zhigalova N.A., Prokhortchouk E.B., Zaraisky A.G. (2020). Cytoskeletal Protein Zyxin Inhibits the Activity of Genes Responsible for Embryonic Stem Cell Status. Cell Rep..

[103] Huang F.K., Han S., Xing B., Huang J., Liu B., Bordeleau F., Reinhart-King C.A., Zhang J.J., Huang X.Y. (2015). Targeted inhibition of fascin function blocks tumour invasion and metastatic colonization. Nat. Commun..

[104] Jiu Y., Lehtimäki J., Tojkander S., Cheng F., Jäälinoja H., Liu X., Varjosalo M., Eriksson J.E., Lappalainen P. (2015). Bidirectional Interplay between Vimentin Intermediate Filaments and Contractile Actin Stress Fibers. Cell Rep..

[105] Thiery J.P., Acloque H., Huang R.Y., Nieto M.A. (2009). Epithelial-mesenchymal transitions in development and disease. Cell.

[106] Zhang X., Pei Z., Ji C., Zhang X., Xu J., Wang J., Jimenez-Lopez J.C. (2017). Novel Insights into the Role of the Cytoskeleton in Cancer. Cytoskeleton—Structure, Dynamics, Function and Disease.

[107] Yamasaki T., Seki N., Yamada Y., Yoshino H., Hidaka H., Chiyomaru T., Nohata N., Kinoshita T., Nakagawa M., Enokida H. (2012). Tumor suppressive microRNA-138 contributes to cell migration and invasion through its targeting of vimentin in renal cell carcinoma. Int. J. Oncol..

[108] Kellogg E.H., Hejab N.M.A., Howes S., Northcote P., Miller J.H., Díaz J.F., Downing K.H., Nogales E. (2017). Insights into the distinct mechanisms of action of taxane and non-taxane microtubule stabilizers from cryo-EM structures. J. Mol. Biol..

[109] Shoji K., Ohashi K., Sampei K., Oikawa M., Mizuno K. (2012). Cytochalasin D acts as an inhibitor of the actin-cofilin interaction. Biochem. Biophys. Res. Commun..

[110] Ethier R.C., Simmons C.A. (2008). Introductory Biomechanics: From Cells to Organisms.

[111] Su X., Zhang L., Kang H., Zhang B., Bao G., Wang J. (2019). Mechanical, nanomorphological and biological reconstruction of early stage apoptosis in HeLa cells induced by cytochalasin B. Oncol. Rep..

[112] Runel G., Lopez-Ramirez N., Chlasta J., Masse I. (2021). Biomechanical properties of cancer cells. Cells.

[113] Lekka M., Laidler P., Ignacak J., Łabedz M., Lekki J., Struszczyk H., Stachura Z., Hrynkiewicz A.Z. (2001). The effect of chitosan on stiffness and glycolytic activity of human bladder cells. Biochim. Biophys. Acta.

[114] Ramos J.R., Pabijan J., Garcia R., Lekka M. (2014). The softening of human bladder cancer cells happens at an early stage of the malignancy process. Beilstein J. Nanotechnol..

[115] Lodish H., Berk A., Matsudaira P., Kaiser C.A., Krieger M., Scott M.P., Zipursky L.S., Darnell J. (2004). Molecular Cell Biology.

[116] Karp G. (2010). Cell and Molecular Biology: Concepts and Experiments.

[117] Neurohr G.E., Amon A. (2020). Relevance and regulation of cell density. Trends Cell Biol..

[118] Molines A.T., Lemière J., Gazzola M., Steinmark I.E., Edrington C.H., Hsu C.T., Real-Calderon P., Suhling K., Goshima G., Holt L.J. (2022). Physical properties of the cytoplasm modulate the rates of microtubule polymerization and depolymerization. Dev. Cell.

[119] Luby-Phelps K., Taylor D.L., Lanni F. (1986). Probing the structure of cytoplasm. J. Cell Biol..

[120] Moeendarbary E., Valon L., Fritzsche M., Harris A.R., Moulding D.A., Thrasher A.J., Stride E., Mahadevan L., Charras G.T. (2013). The cytoplasm of living cells behaves as a poroelastic material. Nat. Mater..

[121] Copos C.A., Guy R.D. (2018). A porous viscoelastic model for the cell cytoskeleton. ANZIAM J..

[122] Ong M.S., Deng S., Halim C.E., Cai W., Tan T.Z., Huang R.Y., Sethi G., Hooi S.C., Kumar A.P., Yap C.T. (2020). Cytoskeletal Proteins in Cancer and Intracellular Stress: A Therapeutic Perspective. Cancers.

[123] Biot M.A. (1941). General theory of three-dimensional consolidation. J. Appl. Phys..

[124] Holsbeeks I., Lagatie O., Van Nuland A., Van de Velde S., Thevelein J.M. (2004). The eukaryotic plasma membrane as a nutrient-sensing device. Trends Biochem. Sci..

[125] Mow V.C., Guilak F., Tran-Son-Tay R., Hochmuth R.M. (1994). Cell Mechanics and Cellular Engineering.

[126] Dias C., Nylandsted J. (2021). Plasma membrane integrity in health and disease: Significance and therapeutic potential. Cell Discov..

[127] Barceló-Coblijn G., Martin M.L., de Almeida R.F., Noguera-Salvà M.A., Marcilla-Etxenike A., Guardiola-Serrano F., Lüth A., Kleuser B., Halver J.E., Escribá P.V. (2011). Sphingomyelin and sphingomyelin synthase (SMS) in the malignant transformation of glioma cells and in 2-hydroxyoleic acid therapy. Proc. Natl. Acad. Sci. USA.

[128] Brochard J., Lennon F. (1975). Frequency spectrum of the flicker phenomenon in erythrocytes. J. Phys..

[129] Sauer R.A. (2017). On the computational modeling of lipid bilayers using thin-shell theory. The Role of Mechanics in the Study of Lipid Bilayers.

[130] Rädler J.O., Feder T.J., Strey H.H., Sackmann E. (1995). Fluctuation analysis of tension-controlled undulation forces between giant vesicles and solid substrates. Phys. Rev. E Stat. Phys. Plasmas Fluids Relat. Interdiscip. Topics.

[131] Friedl P., Wolf K. (2010). Plasticity of cell migration: A multiscale tuning model. J. Cell Biol..

[132] Stillwell W. (2016). An Introduction to Biological Membranes: Composition, Structure and Function.

[133] Guck J., Schinkinger S., Lincoln B., Wottawah F., Ebert S., Romeyke M., Lenz D., Erickson H.M., Ananthakrishnan R., Mitchell D. (2005). Optical deformability as an inherent cell marker for testing malignant transformation and metastatic competence. Biophys. J..

[134] Händel C., Schmidt S., Schiller J., Dietrich U., Möhn T., Kießling T., Pawlizak S., Fritsch A., Horn L.C., Briest S. (2015). Cell membrane softening in human breast and cervical cancer cells. New J. Phys..

[135] Gracia R., Bezlyepkina N., Knorr R., Lipowsky R., Dimova R. (2010). Effect of cholesterol on the rigidity of saturated and unsaturated membranes: Fluctuation and electrodeformation analysis of giant vesicles. Soft Matter.

[136] Goetz R.J., Gompper G., Lipowsky R. (1999). Mobility and elasticity of self-assembled membranes. Phys. Rev. Lett..

[137] Jamali Y., Azimi M., Mofrad M.R. (2010). A sub-cellular viscoelastic model for cell population mechanics. PLoS ONE.

[138] Sandersius S.A., Newman T.J. (2008). Modeling cell rheology with the subcellular element model. Phys. Biol..

[139] Van Liedekerke P., Buttenschön A., Drasdo D., Cerrolaza M., Shefelbine S.J., Garzon-Alvarado D. (2018). Off-Lattice Agent-Based Models for Cell and Tumor Growth: Numerical Methods, Implementation, and Applications. Numerical Methods and Advanced Simulation in Biomechanics and Biological Processes.

[140] Chiodoni C., Di Martino M.T., Zazzeroni F., Caraglia M., Donadelli M., Meschini S., Leonetti C., Scotlandi K. (2019). Cell communication and signaling: How to turn bad language into positive one. J. Exp. Clin. Cancer Res..

[141] Beckmann A., Hainz N., Tschernig T., Meier C. (2019). Facets of Communication: Gap Junction Ultrastructure and Function in Cancer Stem Cells and Tumor Cells. Cancers.

[142] Ramilowski J.A., Goldberg T., Harshbarger J., Kloppmann E., Lizio M., Satagopam V.P., Itoh M., Kawaji H., Carninci P., Rost B. (2015). A draft network of ligand-receptor-mediated multicellular signaling in human. Nat. Commun..

[143] Minc N., Burgess D., Chang F. (2009). Influence of cell geometry on division-plane positioning. Cell.

[144] Drasdo D., Hoehme S. (2012). A cell-based simulation software for multi-cellular systems. Bioinformatics.

[145] Friedl P., Wolf K., Lammerding J. (2011). Nuclear mechanics during cell migration. Curr. Opin. Cell Biol..

[146] Dahl K.N., Ribeiro A.J.S., Lammerding J. (2008). Nuclear shape, mechanics, and mechanotransduction. Circ. Res..

[147] Lammerding J. (2011). Mechanics of the nucleus. Compr. Physiol..

[148] Bustamante C., Marko J.F., Siggia E.D., Smith S. (1994). Entropic elasticity of λ-phage DNA. Science.

[149] Marko J.F., Siggia E.D. (1995). Stretching DNA. Macromol..

[150] Suresh S. (2007). Biomechanics and biophysics of cancer cells. Acta Biomater..

[151] Cross S.E., Jin Y.S., Rao J., Gimzewski J.K. (2007). Nanomechanical analysis of cells from cancer patients. Nat. Nanotechnol..

[152] Gardel M.L., Shin J.H., MacKintosh F.C., Mahadevan L., Matsudaira P., Weitz D.A. (2004). Elastic behavior of cross-linked and bundled actin networks. Science.

[153] Storm C., Pastore J.J., MacKintosh F.C., Lubensky T.C., Janmey P.A. (2005). Nonlinear elasticity in biological gels. Nature.

[154] Petrie R.J., Koo H., Yamada K.M. (2014). Generation of compartmentalized pressure by a nuclear piston governs cell motility in a 3D matrix. Science.

[155] Charras G., Sahai E. (2014). Physical influences of the extracellular environment on cell migration. Nat. Rev. Mol. Cell Biol..

